# Sleep, internalizing symptoms, and health-related quality of life in children with neurodevelopmental disorders: a cross-sectional analysis of cohort data from three research programs in Canada

**DOI:** 10.3389/frsle.2023.1224610

**Published:** 2023-08-24

**Authors:** Patrick G. McPhee, Stelios Georgiades, Andrea Andrade, Penny V. Corkum, Anthony L. Vaccarino, Heena Cheema, Rachel Chepesiuk, Alana Iaboni, Jan Willem Gorter, The OBI Cross-integrated Discovery Program Sleep Team

**Affiliations:** ^1^Department of Pediatrics, McMaster University, Hamilton, ON, Canada; ^2^CanChild Centre for Childhood Disability Research, McMaster University, Hamilton, ON, Canada; ^3^School of Rehabilitation Science, McMaster University, Hamilton, ON, Canada; ^4^Department of Psychiatry and Behavioural Neurosciences, McMaster University, Hamilton, ON, Canada; ^5^Department of Paediatrics, Schulich School of Medicine and Dentistry, Western University, London, ON, Canada; ^6^Department of Psychology and Neuroscience, Dalhousie University, Halifax, NS, Canada; ^7^Department of Psychiatry, Dalhousie University, Halifax, NS, Canada; ^8^Department of Pediatrics, IWK Health, Halifax, NS, Canada; ^9^Indoc Research, Toronto, ON, Canada; ^10^Ontario Brain Institute, Toronto, ON, Canada; ^11^Holland Bloorview Kids Rehabilitation Hospital, Toronto, ON, Canada; ^12^Center of Excellence for Rehabilitation Medicine, UMC Utrecht Brain Center, University Medical Center Utrecht, Utrecht University and De Hoogstraat Rehabilitation, Utrecht, Netherlands; ^13^Department of Rehabilitation, Physical Therapy Science and Sports, UMC Utrecht Brain Center, University Medical Center Utrecht, Utrecht, Netherlands

**Keywords:** child, neurodevelopmental disorder, sleep, quality of life, internalizing symptoms

## Abstract

**Objective:**

The objectives of this study were to determine rates of sleep disturbances in children with neurodevelopmental disorders (NDDs) within and across disorders and compared to typically developing (TD) children and to describe differences above and below the clinical cut-off for sleep disturbances. In addition, we explored the associations between demographic variables, severity of disorder, sleep disturbances, internalizing symptoms, and health-related quality of life (HRQOL) in children with NDDs.

**Method:**

We conducted cross-sectional data analyses of an existing database with community-dwelling children with NDDs (*n* = 1438) and TD children (*n* = 140) aged 4–12 years. Parent-reported measures on sleep disturbances using the Children's Sleep Habits Questionnaire (CSHQ), internalizing symptoms using the Revised Children's Anxiety and Depression Scale, and HRQOL using the KINDL-R were assessed. Hierarchical linear regression examined the associations between demographic variables, severity of disorder, sleep disturbances, internalizing symptoms, and HRQOL in children with NDDs.

**Results:**

Children with NDDs (8.5 ± 2.1 years, 69.9% M) had significantly greater total sleep disturbance index (TSDI) than TD children [(8.6 ± 2.3 years, 60.0% M) (mean difference = 6.88 [95% CI 5.37, 8.40]; *p* < 0.001) (*n* = 838 NDDs (58.3%); *n* = 120 TD (86.7%)]. Children with severe NDDs reported significantly greater TSDI above the clinical cut-off (i.e., ≥41; CSHQ) than those with less severe NDDs (*p* < 0.001). Internalizing symptoms (β = −0.082 [95% CI −0.144, −0.019]; *p* = 0.011) and TSDI (β = −0.226 [95% CI −0.380, −0.073]; *p* = 0.004) were significantly associated with HRQOL in children with NDDs.

**Conclusion:**

Surveillance and management of sleep and internalizing symptoms are needed to improve HRQOL in children with NDDs. Commonalities in sleep disturbances for children with NDDs support transdiagnostic interventions to treat sleep.

## Introduction

Children and young people with disabilities have difficulties meeting the guidelines for healthy sleep (Shelton and Malow, 2021) and sleep less than typically developing (TD) children (Humphreys et al., 2014). A consensus statement from the American Academy of Sleep Medicine recommended the following age-appropriate sleep durations for children: 10–13 h (3-to-5-year-olds); 9–12 h (6-to-12-year-olds); and 8–10 h (13-to-18-year-olds) (Paruthi et al., 2016). Adhering to these recommendations was associated with improved attention, behavior, memory, quality of life, mental health, and physical functioning (Paruthi et al., 2016). Despite sleep recommendations being for *all* children, parents of children and young people with disabilities indicated that sleep guidelines were not inclusive or compatible with the functional abilities of children with disabilities (Handler et al., 2019).

Children with neurodevelopmental disorders (NDDs) can experience sleep problems, including onset, maintenance, and early morning awakenings. The Diagnostic and Statistical Manual of Mental Disorders Fifth Edition (DSM-V) defined neurodevelopmental disorders as a group of conditions with onset in the developmental period. Neurodevelopmental disorders in the DSM-V include but are not limited to intellectual disability (ID), communication disorders, autism spectrum disorder (ASD), attention-deficit/hyperactivity disorder (ADHD), motor disorders, and other neurodevelopmental disorders. A recent systematic review reported that the global prevalence of NDDs according to the DSM-V ranged from ~0.6 to 17% (Francés et al., 2022). For children with a diagnosed NDD, the relationship between sleep and disability may be bidirectional: sleep problems may affect brain functioning (e.g., poor sleep may lead to difficulties in functioning at school during the day), while the (neurodevelopmental) disability may contribute to poor sleep (Halstead et al., 2021). In children with NDDs, the prevalence of reported sleep problems ranged from 42 to 86% (Urquhart et al., 2016; Verschuren et al., 2017; Díaz-Román et al., 2018), which was greater than sleep problems in TD children ranging from 20 to 23% (Lewien et al., 2021).

The sleep problems are substantial and vary within and across NDDs. In children with cerebral palsy (CP), bedtime behaviour and sleep onset, and anxiety around sleep, were significantly higher compared to TD children (Lélis et al., 2016). The prevalence of sleep problems in children with CP was inversely associated with gross motor function ability, and parents reported pain as a significant contributor to sleep disturbances (Verschuren et al., 2017). In children with epilepsy, sleep problems can be due to seizure burden, antiepileptic therapies, and type of epilepsy. This effect is bidirectional given that poor seizure control affects sleep, and poor sleep can worsen the seizure burden (Rodriguez, 2007). In children with ADHD, sleep problems included bedtime resistance, insomnia, and daytime sleepiness (Yoon et al., 2012). More than 50% of children with ASD experienced one or more sleep problems (Hodge et al., 2014), and sleep problems are a co-existing symptom of ASD (Mayes and Calhoun, 2009). In children with anxiety disorders, such as obsessive-compulsive disorders (OCD), sleep problems were highly prevalent (i.e., at least 50%) (Storch et al., 2008) and include increased sleep onset and reduced sleep duration. In children with ID, sleep duration and sleep quality were reduced compared to those without intellectual disabilities (Surtees et al., 2018). However, these sleep problems are not uniformly distributed across NDDs (Halstead et al., 2021). As such, a better understanding of the rates and common occurrences of sleep problems within and across NDDs might provide an opportunity for a generic intervention based on the sleep problem, in addition to disorder-specific treatment.

Sleep problems in children with NDDs can affect other domains of health, such as health-related quality of life (HRQOL) and internalizing symptoms of anxiety and depression (hereafter known as internalizing symptoms) (Rzepecka et al., 2011; Verschuren et al., 2017). The relationships between sleep problems, HRQOL, severity of disorder, and internalizing symptoms across multiple NDDs are unknown. Indeed, children with NDDs with sleep disturbances experienced concomitant anxiety and depression (Kamara and Beauchaine, 2020). The complexity and interplay between NDD, sleep disturbances, and internalizing symptoms complicate a treatment plan (Chorney et al., 2008). A shift in research suggested a transdiagnostic approach (i.e., across multiple NDDs) to treating sleep problems in children with NDDs (Rigney et al., 2018). Understanding the association between sleep disturbances and HRQOL while considering the influence of internalizing symptoms might help inform personalized/tailored interventions and improve clinical care for children with NDDs.

The objectives of this study were (1) to determine rates of sleep disturbances in children with NDDs within and across disorders compared to TD children; (2) to describe differences above and below the clinical cut-off for sleep disturbances in children with NDDs; and (3) to explore the associations between demographic variables, severity of disorder, sleep disturbances, internalizing symptoms, and HRQOL in children with NDDs.

## Materials and methods

This cross-sectional analysis utilized secondary cohort data pertaining to children with clinically diagnosed NDDs inclusive of the following disorders: ASD, ADHD, OCD, ID, CP, and epilepsy (Bozzi et al., 2012; American Psychiatric Association, 2013). All 4–12-year-old children with NDDs at baseline entry into The Province of Ontario Neurodevelopmental Disorders (POND) Network (https://pond-network.ca) (2014–2019), The Childhood Cerebral Palsy Integrated Neuroscience Discovery Network (CP-NET) (http://cpnet.canchild.ca) (2011–2020), and The Epilepsy Research Program (EpLink) (https://eplink.ca) (2013–2018) studies were eligible, and participants consented to have their results used in research. POND also collected information from TD children to serve as a comparison group. These three programs are Integrated Discovery Programs (IDPs) funded by the Ontario Brain Institute (OBI) (https://braininstitute.ca). Details of each of the three programs that comprise the IDPs are described in Supplementary Table 1. The NDDs included in this secondary data analysis were selected based on the availability in each of the IDPs. The Hamilton Integrated Research Ethics Board provided approval for this study (REB #12801).

### Brain-CODE

Data from each IDP were collected locally and then transferred and stored securely in Brain-CODE, OBI's neuroinformatics platform (https://www.braincode.ca/) (Vaccarino et al., 2018).

### Common data elements

OBI implemented a set of standardized assessments to facilitate sharing of patient-level data, known as the Common Data Elements (CDEs). These CDEs include demographical and clinical variables and were agreed upon following a rigorous consensus procedure: researchers from OBI's five IDPs were invited to complete an online survey to rate the importance of collecting demographic and clinical variables; consensus levels of >70% among researchers were considered in order to include the variable as a CDE (Vaccarino et al., 2022). Participants in each study completed the OBI medical history form and demographic form to gather information on age, sex, ethnicity, household income, and parent/caregiver education level. Clinical CDEs included the Children's Sleep Habits Questionnaire (CSHQ) (Owens et al., 2000), the KINDL-R (Ravens-Sieberer and Bullinger, 1998) to assess HRQOL, and the Revised Children's Anxiety and Depression Scale (RCADS) (Chorpita et al., 2000). Severity of disorder was defined via clinical thresholds for the appropriate outcome measures (i.e., severity of ASD using the Social Communication Questionnaire (SCQ) (≥15 = more severe) (Eaves et al., 2006); severity of ADHD using the SWAN Rating Scale (total of 6 or more points in either inattentive or hyperactive impulsive subscales = more severe) (Swanson et al., 2012); severity of OCD using the Toronto-Obsessive-Compulsive Scale (>0 = more severe) (Park et al., 2016); severity of CP using the Gross Motor Function Classification System (GMFCS) (≥3 = more severe) (Palisano et al., 2008); and severity of epilepsy by use of medication (≥2 anti-epileptic drugs = more severe) (Kwan et al., 2010). However, severity of ID was not classified.

### Children's sleep habits questionnaire

The CSHQ is a retrospective, 45-item parent-reported questionnaire that examines sleep behaviors in children during the previous week (Owens et al., 2000). It contains items that reflect eight sleep domains related to clinical sleep complaints in children: bedtime resistance; sleep onset delay; sleep duration; sleep anxiety; night wakings; parasomnias; sleep-disordered breathing; and daytime sleepiness. The ratings for 33 items are totaled to generate a Total Sleep Disturbance Index (TSDI); a score ≥41 is indicative of a pediatric sleep disorder (Owens et al., 2000). The CSHQ has an acceptable internal consistency of 0.78 and test-retest reliability of 0.62 to 0.79 in a clinical sample of children. Using the threshold score of 41, the sensitivity and specificity of the CSHQ were calculated at 0.80 and 0.72, respectively. The CSHQ has the ability to differentiate sleep disorders between community and clinical samples of children (Owens et al., 2000).

### KINDL-R

The KINDL-R is an instrument for assessing HRQOL in children and adolescents aged 3 years and older and consists of 24 items comprising the six subscales of physical wellbeing, emotional wellbeing, self-esteem, family, friends, and everyday functioning. Parent versions were used for this study. Items can be totaled, reverse-scored and transformed to a 0 to 100 range, with higher values indicating better quality of life. Internal consistency for all subscale scores of the KINDL-R in chronically ill children achieved a Cronbach's alpha of at least 0.76 (range 0.76–0.89); the Cronbach's alpha for total score was 0.95 (Ravens-Sieberer and Bullinger, 1998).

### Revised children's anxiety and depression scale

The RCADS (Chorpita et al., 2000) is a 47-item self-report outcome measure that assesses the presence and frequency of anxiety and depressive symptoms in children 8–18 years old. The subscales correspond to separation anxiety disorder (7 items), social phobia (9 items), generalized anxiety disorder (6 items), panic disorder (9 items), obsessive-compulsive disorder (6 items), and major depressive disorder (10 items). The RCADS generates a total anxiety score from the five anxiety subscales (total score range 0–111), and a total internalizing score from all six subscales (total score range 0–141). Internal consistency in a clinical sample of 513 children revealed that the subscales achieved a minimum Cronbach's alpha of 0.78 (range 0.78–0.87) (Chorpita et al., 2005).

### Statistical analysis

Participant demographics, including age, ethnicity, severity, primary caregiver education level, and household income, were expressed as mean (standard deviation) for continuous variables and frequency (percentage) for categorical variables at baseline enrollment into the research programs. Means (standard deviations) were reported for the CSHQ, KINDL-R, and RCADS.

For the first objective, all eight subscales of the CSHQ were calculated, followed by totaling the subscale scores using 33 items, to derive the TSDI (Owens et al., 2000). TSDI scores and the eight subscales were compared between children with NDDs and TD children using independent samples *t*-tests. Between-disorder comparisons were made using ANOVA, and pairwise comparisons were performed and multiple comparisons were adjusted using Bonferroni correction if the between-disorder comparisons were significant. For the second objective, children with NDDs were dichotomized based on meeting the clinical cut-off (≥41) for the CSHQ (TSDI), and comparisons were made for age, sex, severity, KINDL-R (total score), and RCADS [(total internalizing score (anxiety and depression scores combined)] using independent samples *t*-tests (continuous variables) or the chi-squared tests (dichotomous variables). The third objective was analyzed using unadjusted linear regression analysis: age, sex, severity, RCADS total internalizing score, and TSDI were the independent variables, and the KINDL-R total score was the dependent variable in separate univariable models. Hierarchical multivariable linear regression analysis was used to investigate associations between demographic variables (age, sex; step 1), severity (step 2), internalizing symptoms (step 3), and TSDI (step 4) with KINDL-R total score. Multicollinearity was assessed using the variance inflation factor (VIF). Analyses including the RCADS were limited to children ≥8 years old. Statistical analyses were performed using the STATA statistical software package, version 16.1. Statistical tests for final models were performed with two-sided tests at the alpha error probability of 0.05.

### Missing data

**Figure 1:**
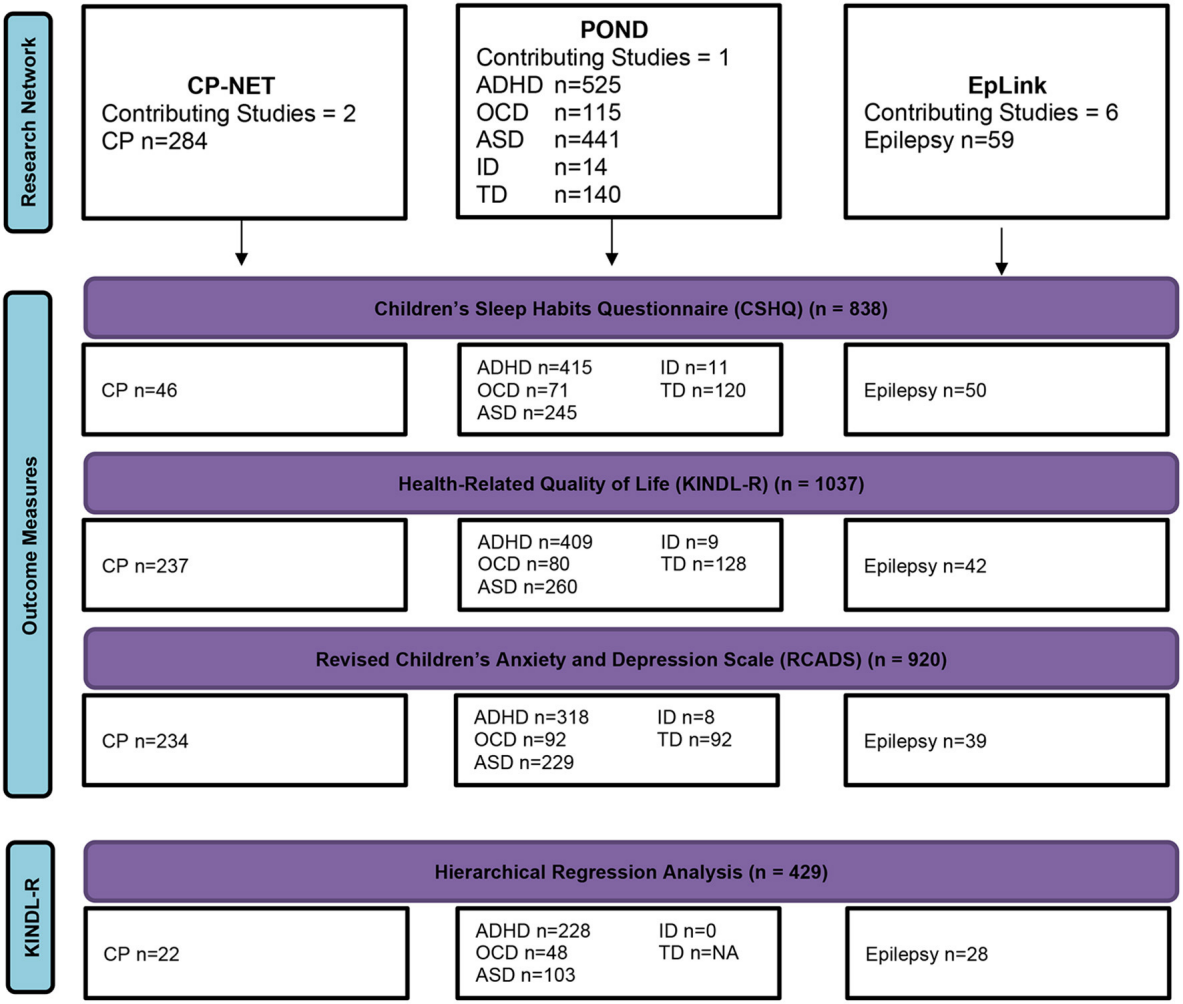
A flow diagram of complete participant data. ADHD, attention-deficit/hyperactivity disorder; ASD, autism spectrum disorder; CP, cerebral palsy; ID, intellectual disability; OCD, obsessive-compulsive disorder; TD, typically developing.

Participants were excluded from the analyses by listwise deletion. A sensitivity analysis was performed between children with TSDI data and those with missing TSDI data to test for differences in the RCADS total internalizing score and the KINDL-R total score. Figure 1 shows a flow diagram of the participant numbers for each disorder for each outcome measure and for multivariable regression analyses.

### Role of the funding source

The study design, analyses, results, and interpretation of data are those of the authors and were not influenced by the Ontario Brain Institute.

## Results

A total of 1,438 children aged 4 to 12 years with a diagnosed NDD (mean age 8.46 years, 69.9% male) met the inclusion criteria for this study. Participants' demographic information and descriptive statistics for the CSHQ, KINDL-R, and RCADS are reported in Table 1. Post-hoc power analysis calculated 98% power (calculated effect size f = 0.33; alpha 0.05; total sample size = 958; 7 groups) to detect differences in sleep disturbances between children with NDDs (combined and individual NDDs) and TD children.

**Table 1:** Participant demographic and outcome characteristics.

	**Total NDDs (*n =* 1,438)**	**TD (*n =* 140)**	**ADHD (*n =* 525)**	**ASD (*n =* 441)**	**CP (*n =* 284)**	**OCD (*n =* 115)**	**Epilepsy (*n =* 59)**	**ID (*n =* 14)**
Mean age – yrs (SD)	8.46 (2.12)	8.57 (2.27)	8.80 (1.93)	8.41 (2.46)	7.27 (1.16)	10.04 (1.58)	8.26 (2.29)	8.07 (2.30)
**Sex – n (%)**								
Male	1,002 (69.9)	84 (60.0)	374 (71.2)	352 (79.8)	163 (58.0)	67 (58.3)	35 (60.3)	11 (78.6)
Female	432 (30.1)	56 (40.0)	151 (28.8)	89 (20.2)	118 (42.0)	48 (41.7)	23 (39.7)	3 (21.4)
**Ethnicity – n (%)**								
Caucasian	894 (73.3)	96 (70.6)	336 (80.4)	279 (75.0)	146 (52.5)	76 (90.5)	45 (81.8)	12 (100.0)
Other	325 (26.7)	40 (29.4)	82 (19.6)	93 (25.0)	132 (47.5)	8 (9.5)	10 (18.2)	0 (0.0)
**Education level – n (%)**								
< High school	51 (5.5)	1 (0.7)	5 (2.8)	15 (4.2)	27 (9.7)	2 (4.3)	0 (0)	2 (20.0)
High school diploma	72 (7.8)	5 (3.7)	17 (9.7)	24 (6.7)	27 (9.7)	0 (0)	3 (5.4)	1 (10.0)
University/College	631 (68.4)	78 (57.8)	126 (71.6)	252 (70.4)	172 (61.7)	32 (68.1)	44 (78.6)	5 (50.0)
Master's degree	102 (11.1)	30 (22.2)	19 (10.8)	40 (11.2)	28 (10.0)	9 (19.2)	5 (8.9)	1 (10.0)
Doctoral/Professional	66 (7.2)	21 (15.6)	9 (5.1)	27 (7.5)	21 (7.5)	4 (8.5)	4 (7.1)	1 (10.0)
**Income – n (%)**								
< $50,000	211 (26.7)	13 (10.7)	48 (29.3)	78 (26.1)	73 (31.1)	5 (13.5)	4 (8.5)	3 (37.5)
$50,000–$99,999	240 (30.4)	23 (18.9)	44 (26.8)	97 (32.4)	66 (28.1)	14 (37.8)	16 (34.0)	3 (37.5)
$100,000–$199,999	252 (31.9)	58 (47.5)	55 (33.5)	100 (33.4)	62 (26.4)	15 (40.5)	20 (42.6)	0 (0)
>$200,000	87 (11.0)	28 (23.0)	17 (10.4)	24 (8.0)	34 (14.5)	3 (8.1)	7 (14.9)	2 (25.0)
**Severity – n (%)**								
Less severe	457 (33.4)	NA	153 (30.4)	94 (22.8)	164 (58.6)	18 (15.7)	28 (50)	NA
More severe	910 (66.6)	NA	350 (69.6)	319 (77.2)	116 (41.4)	97 (84.3)	28 (50)	NA
**CSHQ (mean, SD)**								
TSDI	47.45 (8.19)^a^ *n =* 838	40.57 (5.64) *n =* 120	48.20 (8.23)^ab^ *n =* 415	46.63 (8.24)^a^ *n =* 245	44.41 (7.00) *n =* 46	48.49 (8.82)^a^ *n =* 71	46.46 (6.57)^a^ *n =* 50	48.27 (8.84)^a^ *n =* 11
Bedtime resistance	8.41 (2.52)^a^ *n =* 916	7.24 (1.66) *n =* 128	8.49 (2.55)^a^ *n =* 431	8.40 (2.60)^a^ *n =* 295	8.31 (2.41) *n =* 49	8.33 (2.33)^a^ *n =* 76	8.06 (2.19) *n =* 53	8.58 (2.57) *n =* 12
Sleep onset delay	1.72 (0.81)^a^ *n =* 1,230	1.33 (0.56) *n =* 134	1.90 (0.86)^ab^ *n =* 449	1.75 (0.79)^ab^ *n =* 361	1.36 (0.63) *n =* 270	1.98 (0.76)^ab^ *n =* 83	1.51 (0.72) *n =* 55	1.58 (0.67) *n =* 12
Sleep duration	4.46 (1.71)^a^ *n =* 992	3.56 (1.06) *n =* 130	4.66 (1.72)^ab^ *n =* 446	4.40 (1.76)^ab^ *n =* 346	3.56 (1.00) *n =* 52	4.51 (1.68)^ab^ *n =* 83	3.98 (1.57) *n =* 53	4.25 (1.48) *n =* 12
Sleep anxiety	6.40 (2.21)^a^ *n =* 955	5.10 (1.44) *n =* 129	6.37 (2.21)^a^ *n =* 443	6.40 (2.15)^a^ *n =* 319	6.47 (2.36)^a^ *n =* 49	6.60 (2.33)^a^ *n =* 81	6.12 (2.06) *n =* 51	6.92 (2.78) *n =* 12
Night wakings	4.36 (1.61)^a^ *n =* 1,168	3.71 (1.23) *n =* 129	4.24 (1.62)^a^ *n =* 442	4.51 (1.63)^a^ *n =* 329	4.39 (1.60)^a^ *n =* 256	4.12 (1.34) *n =* 78	4.53 (1.64)^a^ *n =* 51	5.00 (1.95)^a^ *n =* 12
Parasomnias	9.24 (1.96)^a^ *n =* 930	8.11 (1.31) *n =* 126	9.17 (1.95)^a^ *n =* 437	9.37 (2.00)^a^ *n =* 305	8.81 (1.42) *n =* 47	9.33 (2.19)^a^ *n =* 78	9.08 (1.64)^a^ *n =* 51	10.00 (2.41)^a^ *n =* 12
Sleep dis. breath.	3.41 (0.84)^a^ *n =* 950	3.19 (0.54) *n =* 128	3.35 (0.81) *n =* 441	3.48 (0.86)^a^ *n =* 323	3.56 (1.17) *n =* 46	3.32 (0.61) *n =* 79	3.37 (0.70) *n =* 49	4.08 (1.16)^a^ *n =* 12
Daytime sleepiness	12.74 (3.27)^a^ *n =* 961	11.10 (2.61) *n =* 129	13.20 (3.28)^abc^ *n =* 446	12.18 (3.15)^a^ *n =* 326	11.20 (2.31) *n =* 50	13.06 (3.64)^ab^ *n =* 78	13.56 (3.02)^ab^ *n =* 50	11.82 (3.95) *n =* 11
**KINDL-R (mean, SD)**								
KINDL-R total	65.34 (12.65)^a^ *n =* 1,037	72.83 (10.40) *n =* 128	64.44 (13.89)^ab^ *n =* 409	63.60 (12.25)^ab^ *n =* 260	68.78 (10.42) *n =* 237	66.52 (11.24)^a^ *n =* 80	65.33 (13.14)^a^ *n =* 42	55.79 (8.58)^ab^ *n =* 9
**RCADS (mean, SD)**								
Total internalizing score	29.78 (20.62)^a^ *n =* 920	14.74 (10.71) *n =* 92	34.04 (21.20)^ab^ *n =* 318	29.88 (19.37)^ab^ *n =* 229	16.37 (12.36) *n =* 234	48.34 (19.48)^ab^ *n =* 92	32.28 (16.34)^ab^ *n =* 39	24.63 (18.42) *n =* 8

^a^Significantly different from typically developing (TD). ^b^Significantly different from cerebral palsy (CP). ^c^Significantly different from autism spectrum disorder (ASD).

### Objective 1: to determine rates of sleep disturbances across children with NDDs and vs. TD children

Children with NDDs, with all disorders combined, reported significantly greater TSDI scores compared to TD children (MD = 6.88 [95% CI 5.37, 8.40]; *p* < 0.001). Children with ADHD, ASD, OCD, epilepsy, and ID all had significantly greater TSDI compared to TD children, whereas those with CP did not. Importantly, mean TSDI scores for each NDD were above the clinical cut-off value, which was indicative of a pediatric sleep disorder (≥41) (Table 1).

When investigating the CSHQ subscales, Children with NDDs, with all disorders combined, reported greater concerns on all eight subscales compared to TD children, including bedtime resistance (MD = 1.17 [95% CI 0.72, 1.62]; *p* < 0.001), sleep onset delay (MD = 0.39 [95% CI 0.25, 0.53]; *p* < 0.001), sleep duration (MD = 0.90 [95% CI 0.60, 1.20]; *p* < 0.001), sleep anxiety (MD = 1.30 [95% CI 0.91, 1.69]; *p* < 0.001), night wakings (MD = 0.66 [95% CI 0.37, 0.94]; *p* < 0.001), parasomnias (MD = 1.13 [95% CI 0.78, 1.13]; *p* < 0.001), sleep disordered breathing (MD = 0.23 [95% CI 0.08, 0.37]; *p* = 0.003), and daytime sleepiness (MD = 1.64 [95% CI 1.05, 2.30]; *p* < 0.001). Regarding individual NDDs, children with ASD scored significantly higher on every subscale than TD children; children with CP scored higher on sleep anxiety and night wakings subscales only compared to TD children.

There were no significant differences between individual NDDs for bedtime resistance, sleep anxiety, night wakings, parasomnias, and sleep-disordered breathing. Children with CP scored significantly lower than children with ASD, ADHD, and OCD for sleep onset delay and sleep duration. Children with ADHD reported significantly more daytime sleepiness than children with ASD (Table 1).

### Objective 2: to describe differences in children with NDDs above and below the clinical cut-off for sleep disturbances

Among children with NDDs, 78.6% had a TSDI indicative of a pediatric sleep disorder (≥41) compared to 41.6% in TD children (Table 2). Children with NDDs combined and dichotomized as more severe were significantly more likely to report TSDI ≥41 than those that were combined and dichotomized as less severe (*p* < 0.001). Regarding individual NDDs, children with more severe ADHD were significantly more likely to report TSDI ≥41 compared to children with less severe ADHD (*p* < 0.001), while children with less severe CP reported significantly more TSDI ≥41 than children with more severe CP (*p* < 0.05). Children with NDDs combined (*p* < 0.001) and children with ADHD (*p* < 0.001) or CP (*p* < 0.05) with TSDI ≥41 reported significantly lower HRQOL than those with TSDI < 41. Children with NDDs combined (*p* < 0.05) and children with ADHD (*p* < 0.001), ASD (*p* < 0.001), OCD (*p* < 0.05), or epilepsy (*p* < 0.001) with TSDI ≥41 reported significantly higher total internalizing scores than those with TSDI < 41 (Table 2).

**Table 2:** Descriptive characteristics of children with NDDs with Total Sleep Disturbance Index scores above and below clinical cut-off of 41.

	**Total NDDs (*****n** =* **838)**		**TD (*****n** =* **120)**		**ADHD (*****n** =* **415)**		**ASD (*****n** =* **245)**		**CP (*****n** =* **46)**		**OCD (*****n** =* **71)**		**Epilepsy (*****n** =* **50)**		**ID (*****n** =* **11)**	
	≥41 *n =* 659	≤ 40 *n =* 179	≥41 *n =* 50	≤ 40 *n =* 70	≥41 *n =* 340	≤ 40 *n =* 75	≥41 *n =* 178	≤ 40 *n =* 67	≥41 *n =* 31	≤ 40 *n =* 15	≥41 *n =* 60	≤ 40 *n =* 11	≥41 *n =* 42	≤ 40 *n =* 8	≥41 *n =* 8	≤ 40*n =* 3
Mean age – yrs (SD)	8.54 (2.11)	8.68 (2.03)	8.06 (2.32)	8.80 (2.08)	8.61 (1.89)	8.73 (1.87)	8.23 (2.48)	8.48 (2.29)	7.83 (1.51)	7.75 (1.23)	10.02 (1.56)	10.55 (1.44)	7.96 (2.26)	9.28 (1.68)	7.25 (2.12)	8.00 (2.00)
Sex – n (%)																
Male	462 (77.7)	132 (22.3)	32 (43.8)	41 (56.2)	239 (80.5)	58 (19.5)	139 (71.6)	55 (28.4)	16 (69.6)	7 (30.4)	35 (85.4)	6 (14.6)	27 (90.0)	3 (10.0)	6 (66.7)	3 (33.3)
Female	197 (80.7)	47 (19.3)	18 (38.3)	29 (61.7)	101 (85.6)	17 (14.4)	39 (76.5)	12 (23.5)	15 (65.2)	8 (34.8)	25 (83.3)	5 (16.7)	15 (75.0)	5 (25.0)	2 (100.0)	0(0)
Severity – n (%)																
Less severe	172 (72.3)	66^**^ (27.7)	NA	NA	84 (71.8)	33^**^ (28.2)	39 (67.2)	19 (32.8)	24 (77.4)	7^*^ (22.6)	5 (83.3)	1 (16.7)	20 (76.9)	6 (23.1)	NA	NA
More severe	457 (81.0)	107^**^ (19.0)	NA	NA	245 (85.4)	42^**^ (14.6)	131 (74.4)	45 (25.6)	7 (46.7)	8^*^ (53.3)	55 (84.6)	10(15.4)	19 (90.5)	2 (9.5)	NA	NA
KINDL-R (HRQOL) Mean (SD)	63.78 (13.08) *n =* 527	70.17^**^ (12.08) *n =* 138	70.79 (10.07) *n =* 44	75.00^*^ (10.61) *n =* 67	63.12 (13.86) *n =* 286	70.54^**^ (11.56) *n =* 61	62.89 (12.64) *n =* 126	66.69 (13.36) *n =* 46	72.02 (9.50) *n =* 29	78.94^*^ (6.28) *n =* 14	65.29 (10.56) *n =* 50	72.53 (9.72) *n =* 8	64.42 (11.87) *n =* 31	73.66 (9.95) *n =* 7	57.92 (11.19) *n =* 5	55.73 (2.21)*n =* 2
RCADS (total internalizing score) Mean (SD)	37.39 (20.45) *n =* 381	22.69^*^ (19.75) *n =* 114	21.28 (13.68) *n =* 29	11.42^**^ (7.07) *n =* 52	36.86 (20.51) *n =* 199	22.22^**^ (16.37) *n =* 49	33.92 (20.15) *n =* 86	22.37^**^ (15.28) *n =* 40	24.71 (12.95) *n =* 17	13.67 (7.74) *n =* 6	52.92 (18.69) *n =* 48	39.30^*^ (11.55) *n =* 10	34.14 (14.75) *n =* 28	13.50^**^ (12.69) *n =* 6	26.00 (8.66) *n =* 3	15.67 (8.74)*n =* 3

^*^ p < 0.05, ^**^ p < 0.001.

### Objective 3: to explore associations between demographics, internalizing symptoms, sleep, and HRQOL in children with NDDs

Unadjusted regression analysis revealed a significant inverse association between TSDI and HRQOL (β = −0.354 [95% CI −0.473, −0.235]; *p* < 0.001) in the combined sample of children with NDDs. Hierarchical multivariable regression analyses revealed a significant inverse association between NDD severity (more severe) and HRQOL (β =−2.462 [95% CI−4.109, −0.815]; *p* = 0.003), independent of age and sex (step 2; Table 3). The RCADS total internalizing score was significantly inversely associated with HRQOL (β = −0.139 [95% CI −0.185, −0.092]; *p* < 0.001), independent of age, sex, and NDD severity (step 3; Table 3). Notably, there was a significant R^2^ change with the addition of the RCADS. In the final model (step 4), CSHQ TSDI (β = −0.226 [95% CI −0.380, −0.073]; *p* = 0.004) and RCADS (β = −0.082 [95% CI −0.144, −0.019]; *p* = 0.011) were significantly inversely associated with HRQOL, independent of NDD severity, age, and sex (Table 3).

**Table 3:** Results of regression analyses.

**Test (step)**	**Obs**.	**Outcome**	**Predictor**	**Beta (95% CI)**	***P***	**R^2^**	**R^2^ change**	***P* change**
Univariable reg.	1037	KINDL-R	Age (years)	0.024 (-0.355, 0.403)	0.901	0.000		
Univariable reg.	1037	KINDL-R	Sex (Female)	−0.419 (-2.084, 1.246)	0.621	0.000		
Univariable reg.	990	KINDL-R	**Severity (more severe)**	**−2.387 (−4.010**, **−0.764)**	**0.004**	0.008		
Univariable reg.	724	KINDL-R	**RCADS total internalizing score**	**−0.142 (−0.184**, **−0.099)**	**< 0.001**	0.056		
Univariable reg.	665	KINDL-R	**CSHQ TSDI**	**−0.354 (−0.473**, **−0.235)**	**< 0.001**	0.049		
Hierarchical reg. (1)	1037	KINDL-R	Age (years)	0.023 (−0.356, 0.403)	0.904	0.000		
			Sex (Female)	−0.419 (-2.084, 1.247)	0.622			
Hierarchical reg. (2)	990	KINDL-R	Age (years)	0.074 (-0.317, 0.465)	0.711	0.009	0.008	0.004
			Sex (Female)	−0.379 (−2.084, 1.325)	0.662			
			**Severity (more severe)**	**−2.462 (−4.109**, **−0.815)**	**0.003**			
Hierarchical reg. (3)	710	KINDL-R	Age (years)	−0.055 (−0.563, 0.453)	0.832	0.055	0.046	< 0.001
			Sex (Female)	0.824 (-1.059, 2.707)	0.390			
			Severity (more severe)	0.215 (−1.677, 2.107)	0.824			
			**RCADS total internalizing score**	**−0.139 (−0.185**, **−0.092)**	**< 0.001**			
Hierarchical reg. (4)	429	KINDL-R	Age (years)	−0.191 (−1.017, 0.633)	0.648	0.058	0.003	1.000
			Sex (Female)	0.675 (−1.911, 3.260)	0.608			
			Severity (more severe)	0.143 (−2.462, 2.748)	0.914			
			**RCADS total internalizing score**	**−0.082 (−0.144**, **−0.019)**	**0.011**			
			**CSHQ TSDI**.	**−0.226 (−0.380**, **−0.073)**	**0.004**			

Only participants with complete observations were included in the analysis. Bold text indicates a significant (i.e., *p* < 0.05) predictor.

### Sensitivity analysis

Compared to children with NDDs with complete TSDI, those with missing TSDI (*n* = 600) had significantly lower RCADS total internalizing score (MD = −9.14 [95% CI −11.75, −6.53]; *p* < 0.001). There was no difference in HRQOL between those with complete TSDI and those without complete TSDI.

## Discussion

This study sought to understand sleep disturbances in children with NDDs within and across disorders, and compared to those in TD children. We found that children with NDDs as a collective experienced significantly greater rates of sleep disturbances than TD children for TSDI and each of the eight subscales. Children with ADHD, ASD, OCD, epilepsy, or ID, but not CP, experienced significantly greater rates of sleep disturbances for TSDI than TD children. Children with NDDs and having clinical levels indicative of a pediatric sleep disorder were more likely to have higher NDD symptom severity, lower HRQOL, and more internalizing symptoms. We found that greater TSDI score and more internalizing symptoms were each significantly associated with lower HRQOL in children with NDDs, with all disorders combined, independent of age, sex, and severity. These findings highlight the need for assessment and management of sleep and the need to further understand through longitudinal studies how sleep, internalizing symptoms, and HRQOL impact each other in children with NDDs.

This study presents the first combined analysis of differences in sleep disturbances between children with various NDDs and TD children. Our findings complement previous results that showed that sleep disturbances were greater in children with NDDs across some diagnostic groups compared to TD children and add to the existing literature by discovering similarities between CSHQ subscales across diagnoses (Halstead et al., 2021). For example, in this study, the combined group of NDDs had higher reported rates of parasomnias compared to TD children, which was the case for each NDD except CP. Previous research had reported that only children with CP and those with unspecified conditions experienced greater daytime sleepiness than children with ADHD or epilepsy (Halstead et al., 2021). Our study reported that children with CP had less daytime sleepiness than children with ADHD, OCD, and epilepsy. This difference may be due to the smaller sample size (e.g., *n* = 14 CP) of participants seeking sleep services in the previous study (Halstead et al., 2021) and the fact that the majority (67%) of our sample of children with CP had less severity. Additionally, a significant proportion of children with epilepsy in our sample were receiving more than one anti-epileptic drug, which may have negatively impacted daytime sleepiness (Rodriguez, 2007). Moreover, it was possible that participants in the epilepsy cohorts might have overlapping NDDs, such as ASD (Francis et al., 2013), ADHD (Downs et al., 2017), and/or CP (Wallace, 2001), that potentially contributed to their sleep disturbances. Our study adds to the previous literature by including additional disorders and a substantial sample size to address our study objectives. However, only fifteen children with CP in our sample were classified as having greater limitations in motor ability (GMFCS level ≥3), which might explain why more children with CP with less severity scored above the clinical cut-off for sleep disorders. Thus, these relationships need to be tested in children with CP having more severe motor impairment. Children with NDDs as a collective had greater bedtime resistance, sleep anxiety, night wakings, parasomnias, and sleep-disordered breathing compared to TD children, yet these disturbances were not significantly different among individual disorders. These findings support the importance of a hybrid approach to understanding and treating sleep in children with NDDs: the development and implementation of transdiagnostic sleep interventions for children with NDDs (Rigney et al., 2018) while considering a personalized/tailored approach based on disorder-specific factors.

Findings from our study identified that greater severity of disorder, more internalizing symptoms, and more sleep disturbances were associated with lower HRQOL in children with NDDs as a collective, independent of age and sex. Previous evidence discovered that more sleep disturbances were associated with lower quality of life in children with ASD (Malow et al., 2006) and ADHD (Craig et al., 2020). Our findings extend this relationship to include other NDDs. Collectively, children with NDDs scoring above the clinical cut-off for TSDI reported lower HRQOL compared to children who did not report poor sleep disturbances. Interestingly, we observed a similar relationship in TD children; however, HRQOL in TD children above the clinical cut-off for sleep disorder was comparable to HRQOL in children with NDDs below the clinical cut-off. Moreover, internalizing symptoms were associated with decreased HRQOL in children with NDDs. Indeed, internalizing symptoms are common in children with various NDDs: children with ASD, ADHD, epilepsy, or OCD experience high levels of anxiety, depression, and stress compared to TD children (Gothelf et al., 2004; Rzepecka et al., 2011; Jones et al., 2015). These characteristics likely share a similar association with overall HRQOL, which was supported by the significant association between increased internalizing symptoms and decreased HRQOL.

The influence of severity of disorder was significantly associated with HRQOL, independent of age and sex, and increased severity was associated with pediatric sleep disorders in children with NDDs. Research suggests that meeting age-appropriate sleep recommendations in children with NDDs has positive benefits on anxiety and depression, independent of disability severity (Brown et al., 2021). Our findings suggest that children classified as more severe based on the diagnosis have increased sleep disturbances, internalizing symptoms, and lower HRQOL than those with a less severe diagnosis. Together, these findings emphasize the importance of adequate sleep (both quantity and quality) for children with NDDs to reduce internalizing symptoms and improve HRQOL.

Sleep is important for other health indicators, as evidenced by the associations with internalizing symptoms and HRQOL from this study. Recent qualitative research suggests that families of children with NDDs face common barriers related to access to treatment, intensity of treatment, and overall exhaustion. In contrast, education and modular treatments for NDD-specific symptoms were viewed as facilitators for treating sleep in children with NDDs (Tan-MacNeill et al., 2020). Our findings show many commonalities in sleep disturbances for children with NDDs, which can not only educate both families and clinicians on sleep issues (Hulst et al., 2021) but also provide support for transdiagnostic interventions to treat sleep, symptoms of anxiety and depression, and HRQOL in children with NDDs, particularly those with more severe disorders. Good examples of such [transdiagnostic] interventions for children with NDDs and their families include “Better Nights, Better Days”, an online program developed by a team of sleep experts across Canada (https://betternightsbetterdays.ca/) (Corkum et al., 2018), and an online educational module for clinicians and parents on sleep issues in the Netherlands (https://emodules.umcutrecht.nl/module/0392-Slaap-bij-kinderen/story.html).

This study has important limitations. We did not have information on medications, concomitant disorders, or diagnostic overlap for all participants; thus, the impact of medications and multiple disorders on the results is unknown. The secondary data contained parent-reported responses, which may introduce response bias. The prevalence of different sleep disturbances in children with a diagnosed NDD should be interpreted with caution, given the use of a subjective sleep questionnaire, as recent literature suggests that neurodevelopmental disorders are associated with subjective sleepiness without the presence of objective sleepiness (Munkhjargal et al., 2022). Dichotomizing severity using disorder-specific measures, although valid, does not equate to a single severity measure when combining NDDs. Thus, results pertaining to severity should be interpreted cautiously. The significant positive association between TSDI and internalizing symptoms should be interpreted with caution since children with missing TSDI had lower internalizing symptoms than those with TSDI data. While the overall sample of children with NDDs was large, analyses of children with ID or epilepsy should be interpreted with caution compared to those of other children with NDDs, given the small sample size of children with ID or epilepsy in this study. Finally, the cross-sectional data limits our understanding of bidirectional relationships between sleep disturbances, severity, HRQOL, and internalizing symptoms. Future longitudinal study designs with representative samples of each NDD are required to understand these associations while also considering the influence of socioeconomic variables (e.g., household income and parental education level) and the impact of comorbidities and multiple disorders on greater sleep disturbances and HRQOL in this population.

## Conclusions

In conclusion, we found that children with NDDs, within and across disorders, have poorer sleep than TD children. Sleep disturbances and internalizing symptoms have strong inverse associations with HRQOL in children with NDDs as a collective, independent of severity, suggesting the need to develop, test, and implement generic interventions for NDD-general factors while also considering disorder-specific factors tailored to the child's condition and behavior (e.g., internalizing behaviors). These findings should be interpreted with caution due to the limitations of secondary data analysis and limited information available in the existing database on medications, comorbidities, and diagnostic overlap.

## Data availability statement

The original, de-identified data (including study protocol and data dictionaries) will be available through Brain-CODE (www.braincode.ca). Requests to access these datasets should be directed to info@braininstitute.ca.

## Ethics statement

This study conducted secondary analysis of existing datasets, and was reviewed and approved by the Hamilton Integrated Research Ethics Board (REB #12801). When partaking in the previous research programs, the participants' legal guardian/parent provided consent for their data to be used in future research.

## Author contributions

SG and AA collected participant data. PGM, HC, and JWG conceived and developed the study idea. PGM and ALV developed the data analysis plan. PGM and HC verified the underlying data. PGM led the data analysis (with input from AI, ALV, and JWG) and led the manuscript drafting. PGM, SG, AA, PVC, ALV, HC, RC, and AI contributed to data interpretation. All authors critically reviewed, provided feedback on, and approved the final version of the manuscript.

## References

[B1] American Psychiatric Association (2013). Diagnostic and Statistical Manual of Mental Disorders: DSM-5. Washington, DC: APA (2013).

[B2] Bozzi Y. Casarosa S. Caleo M. (2012). Epilepsy as a neurodevelopmental disorder. Front. Psychiatr. 3, 19. 10.3389/fpsyt.2012.00019 22457654 PMC3306997

[B3] Brown D. M. McPhee P. G. Kwan M. Y. Timmons B. W. (2021). Implications of disability severity on 24-hour movement guideline adherence among children with neurodevelopmental disorders in the United States. J. Phys. Activity Health 18, 1325–31. 10.31236/osf.io/sy58b 34548417

[B4] Chorney D. B. Detweiler M. F. Morris T. L. Kuhn B. R. (2008). The interplay of sleep disturbance, anxiety, and depression in children. J. Pediatr. Psychol. 33, 339–348. 10.1093/jpepsy/jsm105 17991689

[B5] Chorpita B. F. Moffitt C. E. Gray J. (2005). Psychometric properties of the revised child anxiety and depression scale in a clinical sample. Behav. Res. Ther. 43, 309–322. 10.1016/j.brat.2004.02.004 15680928

[B6] Chorpita B. F. Yim L. Moffitt C. Umemoto L. A. Francis S. E. (2000). Assessment of symptoms of DSM-IV anxiety and depression in children: a revised child anxiety and depression scale. Behav. Res. Ther. 38, 835–855. 10.1016/S0005-7967(99)00130-8 10937431

[B7] Corkum P. V. Reid G. J. Hall W. A. Godbout R. Stremler R. Weiss S. K.. (2018). Evaluation of an internet-based behavioral intervention to improve psychosocial health outcomes in children with insomnia (Better Nights, Better Days): protocol for a randomized controlled trial. JMIR Res. Protocols. 7, e8348. 10.2196/resprot.8348 29581089 PMC5891669

[B8] Craig S. G. Weiss M. D. Hudec K. L. Gibbins C. (2020). The functional impact of sleep disorders in children with ADHD. J. Attent. Disorders. 24, 499–508. 10.1177/1087054716685840 28093033

[B9] Díaz-Román A. Zhang J. Delorme R. Beggiato A. Cortese S. (2018). Sleep in youth with autism spectrum disorders: systematic review and meta-analysis of subjective and objective studies. Evid. Based Mental Health. 21, 146–154. 10.1136/ebmental-2018-300037 30361331 PMC10270396

[B10] Downs J. Giust J. Dunn D. W. (2017). Considerations for ADHD in the child with epilepsy and the child with migraine. Exp. Rev. Neurother. 17, 861–869. 10.1080/14737175.2017.1360136 28749241

[B11] Eaves L. C. Wingert H. D. Ho H. H. Mickelson E. C. (2006). Screening for autism spectrum disorders with the social communication questionnaire. J. Dev. Behav. Pediatr. 27, S95–S103. 10.1097/00004703-200604002-00007 16685191

[B12] Francés L. Quintero J. Fernández A. Ruiz A. Caules J. Fillon G.. (2022). Current state of knowledge on the prevalence of neurodevelopmental disorders in childhood according to the DSM-5: a systematic review in accordance with the PRISMA criteria. Child Adol. Psychiat. Mental Health. 16, 27. 10.1186/s13034-022-00462-1 35361232 PMC8973738

[B13] Francis A. Msall M. Obringer E. Kelley K. (2013). Children with autism spectrum disorder and epilepsy. Pediatric Annal. 42, e264–e9. 10.3928/00904481-20131122-10 24295159

[B14] Gothelf D. Aharonovsky O. Horesh N. Carty T. Apter A. (2004). Life events and personality factors in children and adolescents with obsessive-compulsive disorder and other anxiety disorders. Compreh. Psychiatry. 45, 192–198. 10.1016/j.comppsych.2004.02.010 15124149

[B15] Halstead E. J. Joyce A. Sullivan E. Tywyn C. Davies K. Jones A.. (2021). Sleep disturbances and patterns in children with neurodevelopmental conditions. Front. Pediatr. 9, 91. 10.3389/fped.2021.637770 33738270 PMC7961155

[B16] Handler L. Tennant E. M. Faulkner G. Latimer-Cheung A. E. (2019). Perceptions of inclusivity: The Canadian 24-hour movement guidelines for children and youth. Adapted Phys. Acti. Q. 36, 1–18. 10.1123/apaq.2017-0190 30525924

[B17] Hodge D. Carollo T. M. Lewin M. Hoffman C. D. Sweeney D. P. (2014). Sleep patterns in children with and without autism spectrum disorders: developmental comparisons. Res. Dev. Disab. 35, 1631–1638. 10.1016/j.ridd.2014.03.037 24780146

[B18] Hulst R. Y. Gorter J. W. Voorman J. M. Kolk E. Van Der Vossen S. Visser-Meily J. M.. (2021). Sleep problems in children with cerebral palsy and their parents. Dev. Med. Child Neurol. 63, 1344–50. 10.1111/dmcn.14920 33990937 PMC8597175

[B19] Humphreys J. S. Gringras P. Blair P. S. Scott N. Henderson J. Fleming P. J.. (2014). Sleep patterns in children with autistic spectrum disorders: a prospective cohort study. Arch. Dis. Childhood. 99, 114–118. 10.1136/archdischild-2013-304083 24061777 PMC3913218

[B20] Jones J. E. Jackson D. C. Chambers K. L. Dabbs K. Hsu D. A. Stafstrom C. E.. (2015). Children with epilepsy and anxiety: subcortical and cortical differences. Epilepsia. 56, 283–290. 10.1111/epi.12832 25580566 PMC4340751

[B21] Kamara D. Beauchaine T. P. A. (2020). Review of sleep disturbances among infants and children with neurodevelopmental disorders. Rev. J. Autism Dev. Disorders. 7, 278–294. 10.1007/s40489-019-00193-8 33344102 PMC7747783

[B22] Kwan P. Arzimanoglou A. Berg A. T. Brodie M. J. Allen Hauser W. Mathern G.. (2010). Definition of Drug Resistant Epilepsy: Consensus Proposal by the Ad Hoc Task Force of the ILAE Commission on Therapeutic Strategies. New York, NY: Wiley Online Library (2010). 10.1111/j.1528-1167.2009.02397.x 19889013

[B23] Lélis A. L. P. Cardoso M. V. L. Hall W. A. (2016). Sleep disorders in children with cerebral palsy: An integrative review. Sleep Med. Rev. 30, 63–71. 10.1016/j.smrv.2015.11.008 26874066

[B24] Lewien C. Genuneit J. Meigen C. Kiess W. Poulain T. (2021). Sleep-related difficulties in healthy children and adolescents. BMC Pediatr. 21, 1–11. 10.1186/s12887-021-02529-y 33593333 PMC7885393

[B25] Malow B. A. Marzec M. L. McGrew S. G. Wang L. Henderson L. M. Stone W. L.. (2006). Characterizing sleep in children with autism spectrum disorders: a multidimensional approach. Sleep. 29, 1563–1571. 10.1093/sleep/29.12.1563 17252887

[B26] Mayes S. D. Calhoun S. L. (2009). Variables related to sleep problems in children with autism. Res. Autism Spectr. Dis. 3, 931–941. 10.1016/j.rasd.2009.04.002

[B27] Munkhjargal O. Oka Y. Tanno S. Shimizu H. Fujino Y. Kira T.. (2022). Discrepancy between subjective and objective sleepiness in adolescents. Sleep Med. 96, 1–7. 10.1016/j.sleep.2022.04.025 35569178

[B28] Owens J. A. Spirito A. McGuinn M. (2000). The children's sleep habits questionnaire (CSHQ): psychometric properties of a survey instrument for school-aged children. Sleep. 23, 1043–1052. 10.1093/sleep/23.8.1d 11145319

[B29] Palisano R. J. Rosenbaum P. Bartlett D. Livingston M. H. (2008). Content validity of the expanded and revised gross motor function classification system. Dev. Med. Child Neurol. 50, 744–750. 10.1111/j.1469-8749.2008.03089.x 18834387

[B30] Park L. S. Burton C. L. Dupuis A. Shan J. Storch E. A. Crosbie J.. (2016). The Toronto obsessive-compulsive scale: psychometrics of a dimensional measure of obsessive-compulsive traits. J. Am. Acad. Child Adol. Psychiatry. 55, 310–318. 10.1016/j.jaac.2016.01.008 27015722

[B31] Paruthi S. Brooks L. J. D'Ambrosio C. Hall W. A. Kotagal S. Lloyd R. M.. (2016). Consensus statement of the American academy of sleep medicine on the recommended amount of sleep for healthy children: methodology and discussion. J. Clin. Sleep Med. 12, 1549–1561. 10.5664/jcsm.6288 27707447 PMC5078711

[B32] Ravens-Sieberer U. Bullinger M. (1998). Assessing health-related quality of life in chronically ill children with the German KINDL: first psychometric and content analytical results. Q. Life Res. 7, 399–407. 10.1023/A:1008853819715 9691720

[B33] Rigney G. Ali N. S. Corkum P. V. Brown C. A. Constantin E. Godbout R.. (2018). A systematic review to explore the feasibility of a behavioural sleep intervention for insomnia in children with neurodevelopmental disorders: a transdiagnostic approach. Sleep Med. Rev. 41, 244–254. 10.1016/j.smrv.2018.03.008 29764710

[B34] Rodriguez A. J. (2007). Pediatric sleep and epilepsy. Curr. Neurol. Neurosci. Rep. 7, 342. 10.1007/s11910-007-0052-0 17618542

[B35] Rzepecka H. McKenzie K. McClure I. Murphy S. (2011). Sleep, anxiety and challenging behaviour in children with intellectual disability and/or autism spectrum disorder. Res. Dev. Disab. 32, 2758–2766. 10.1016/j.ridd.2011.05.034 21700417

[B36] Shelton A. R. Malow B. (2021). Neurodevelopmental disorders commonly presenting with sleep disturbances. Neurotherapeutics. 14, 1–14. 10.1007/s13311-020-00982-8 33403472 PMC8116361

[B37] Storch E. A. Murphy T. K. Lack C. W. Geffken G. R. Jacob M. L. Goodman W. K.. (2008). Sleep-related problems in pediatric obsessive-compulsive disorder. J. Anxiety Disorders. 22, 877–885. 10.1016/j.janxdis.2007.09.003 17951025 PMC2413417

[B38] Surtees A. D. Oliver C. Jones C. A. Evans D. L. Richards C. (2018). Sleep duration and sleep quality in people with and without intellectual disability: a meta-analysis. Sleep Med. Rev. 40, 135–150. 10.1016/j.smrv.2017.11.003 29754933

[B39] Swanson J. M. Schuck S. Porter M. M. Carlson C. Hartman C. A. Sergeant J. A.. (2012). Categorical and dimensional definitions and evaluations of symptoms of ADHD: history of the SNAP and the SWAN rating scales. Int. J. Educa. Psychol. Assessment. 10, 51. 26504617 PMC4618695

[B40] Tan-MacNeill K. M. Smith I. M. Jemcov A. Keeler L. Chorney J. (2020). Barriers and facilitators to treating insomnia in children with autism spectrum disorder and other neurodevelopmental disorders: Parent and health care professional perspectives. Res. Dev. Disab. 107, 103792. 10.1016/j.ridd.2020.103792 33126148

[B41] Urquhart D. S. Kehinde O. O. Mclellan A. E. (2016). Observational pilot study of reported symptoms of obstructive sleep apnoea in children with epilepsy. Dev. Med. Child Neurol. 58, 1063–1068. 10.1111/dmcn.13173 27316368

[B42] Vaccarino A. L. Beaton D. Black S. E. Blier P. Farzan F. Finger E.. (2022). Common data elements to facilitate sharing and re-use of participant-level data: assessment of psychiatric comorbidity across brain disorders. Front. Psychiatr. 65, 816465. 10.3389/fpsyt.2022.816465 35197877 PMC8859302

[B43] Vaccarino A. L. Dharsee M. Strother S. Aldridge D. Arnott S. R. Behan B.. (2018). Brain-CODE: a secure neuroinformatics platform for management, federation, sharing and analysis of multi-dimensional neuroscience data. Front. Neuroinf. 12, 28. 10.3389/fninf.2018.00028 29875648 PMC5974337

[B44] Verschuren O. Gorter J. W. Pritchard-Wiart L. (2017). Sleep: an underemphasized aspect of health and development in neurorehabilitation. Early Hum. Dev. 113, 120–128. 10.1016/j.earlhumdev.2017.07.006 28711232

[B45] Wallace S. J. (2001). Epilepsy in cerebral palsy. Dev. Med. Child Neurol. 43, 713–717. 10.1017/S0012162201001281 11665830

[B46] Yoon S. Y. R. Jain U. Shapiro C. (2012). Sleep in attention-deficit/hyperactivity disorder in children and adults: past, present, and future. Sleep Med. Rev. 16, 371–388. 10.1016/j.smrv.2011.07.001 22033171

