# Supplementary material for: Sleep, internalizing symptoms, and health-related quality of life in children with neurodevelopmental disorders: a cross-sectional analysis of cohort data from three research programs in Canada
Source: Front Sleep. 2023 Aug 24;2:1224610. doi: 10.3389/frsle.2023.1224610 (PMC12713885; doi:10.3389/frsle.2023.1224610)
Supplement: Supplementary file 1 [file Table_1.docx]

**Supplementary Table 1.** Information for Integrated Discovery Programs

| **Integrated Discovery Program** | **Description** | **Neurodevelopmental disorder(s)** | **Data collection** |
| --- | --- | --- | --- |
| POND | POND aims to understand the neurobiology of NDDs and translate these findings into effective treatments | ADHD; ASD; ID; OCD  TD | Baseline data to the POND longitudinal clinical registry:  2014-2019 |
| CP-NET | CP-NET is an Ontario-based neuroscience research network focused on enhancing the lives of individuals with CP and their families | CP | Baseline data from clinical database: 2011-2020 |
| EpLink | EpLink is a collaborative program focused on finding new ways to diagnose, treat and improve the lives of people living with epilepsy | Epilepsy | Baseline data from six studies investigating ketogenic diet, surgery, or HRQOL in pediatric epilepsy: 2013-2018 |

ADHD = attention-deficit/hyperactivity disorder; ASD = autism spectrum disorder; CP = cerebral palsy; CP-NET = Cerebral Palsy Integrated Neuroscience Discovery Network; EpLink = Epilepsy Research Program; HRQOL = health-related quality of life; ID = intellectual disability; OCD = obsessive compulsive disorder; POND = Province of Ontario Neurodevelopmental Disorders; TD = typically developing
